# Trifid epiglottis in a neonate with choanal atresia and hydrometrocolpos: the third reported case and the first symptomatic neonatal presentation

**DOI:** 10.1186/s12887-026-07076-y

**Published:** 2026-05-30

**Authors:** Mahdieh Khorashadizadeh, Armin Doostparast, Mohammad Amin Sabahi, Sara Ghahramani

**Affiliations:** 1 Clinical Research Development Unit of Akbar Hospital, Faculty of Medicine, Mashhad University of Medical Sciences, Mashhad, Iran; 2 Eye Research Center, Mashhad University of Medical Sciences, Mashhad, Iran; 3 Student Research Committee, Faculty of Medicine, Mashhad University of Medical Sciences, Mashhad, Iran

**Keywords:** Trifid epiglottis, Congenital laryngeal anomaly, Choanal atresia, Hydrometrocolpos, Polydactyly

## Abstract

**Background:**

Trifid epiglottis (TE) is an extremely rare congenital laryngeal anomaly. To the best of our knowledge, only two cases have previously been reported in the literature. Both of them have been incidentally detected and associated with polydactyly.

**Case presentation:**

We report the third known case of TE in a two-day-old female neonate who was admitted to the emergency department due to abdominal distension, oliguria, and poor feeding within the first 24 h of life. Examination revealed stridor during crying, left postaxial polydactyly, right syndactyly, and an anteriorly displaced anus. Imaging findings revealed hydrometrocolpos followed by bilateral hydronephrosis. Her distension and oliguria were successfully treated with surgical drainage. Due to persistent stridor, airway evaluation confirmed right choanal atresia and TE. Other evaluations, including echocardiography, spinal ultrasonography, endocrine studies, and brain magnetic resonance imaging (MRI), were normal. The patient remained stable during one year of follow-up.

**Conclusion:**

To the best of our knowledge, this appears to be the first reported symptomatic neonatal case of TE associated with choanal atresia and hydrometrocolpos. It highlights the importance of early diagnosis and comprehensive evaluation of rare congenital laryngeal anomalies in neonates. Reporting such cases is crucial to expand the understanding of their clinical spectrum, guide airway management, and perform multidisciplinary care.

## Introduction

Bifid epiglottis (BE) is a rare congenital anomaly of the larynx that may present with airway obstruction, stridor, respiratory distress, or aspiration. It is frequently associated with syndromic conditions such as Pallister–Hall syndrome and Bardet–Biedl syndrome [1–3]. To the best of our knowledge, an even rarer variant, the trifid epiglottis (TE), has only been reported in two cases. Moreover, both of these cases were associated with polydactyly [4, 5].

The clinical spectrum and syndromic associations of TE remain poorly understood due to its extreme rarity. Reporting additional cases may help clarify its clinical significance and potential associations with other congenital anomalies. Here, we report the third known case of TE, and to the best of our knowledge, the first reported one in a neonate with respiratory presentation. This two-day-old female neonate also exhibited multiple congenital anomalies, including choanal atresia, hydrometrocolpos, postaxial polydactyly, and an anteriorly displaced anus. This report emphasizes TE’s rarity, associated anomalies, and provides a review of the two previously reported cases.

## Case presentation

A two-day-old female neonate, the third child of non-consanguineous parents, was admitted to the emergency department due to abdominal distension and poor feeding that had developed within the first 24 h after birth and was followed by oliguria and lethargy. She was born at term at 38 weeks of gestation via cesarean section due to a previous cesarean delivery. Her birth weight, height, and head circumference were 3400 g, 49 cm, and 34 cm, respectively, all appropriate for her gestational age. The mother, a 30-year-old woman, was Gravida 3 Para 3 (G3P3) with no relevant past medical history, medication use, teratogenic exposures, or complications during pregnancy or labor. Prenatal ultrasound examination, including assessment of amniotic fluid volume, was normal. There was no family history of genetic disorders or congenital anomalies. The APGAR score was 8 out of 9 at both one and five minutes. No pathology was observed on physical examinations immediately after delivery. However, during the next 24 h, she developed abdominal distension and oliguria, followed by poor feeding and lethargy. No vomiting or seizure episodes were reported. She had passed meconium during the meantime.

On day 3 of life, she was admitted, and her vital signs were as follows: oxygen saturation (SpO₂): 90% on room air, respiratory rate: 40 breaths/minute, heart rate: 124 beats/minute and blood pressure: 70/50 mmHg. Despite being lethargic, she was responsive to stimulation. Cardiopulmonary examinations, including auscultation and inspection, were normal. There was only a mild intercostal retraction, stridor and cyanosis during crying. Abdominal examination revealed marked abdominal distention without tenderness, localized bulging, or palpable hepatosplenomegaly.

**Fig. 1:**
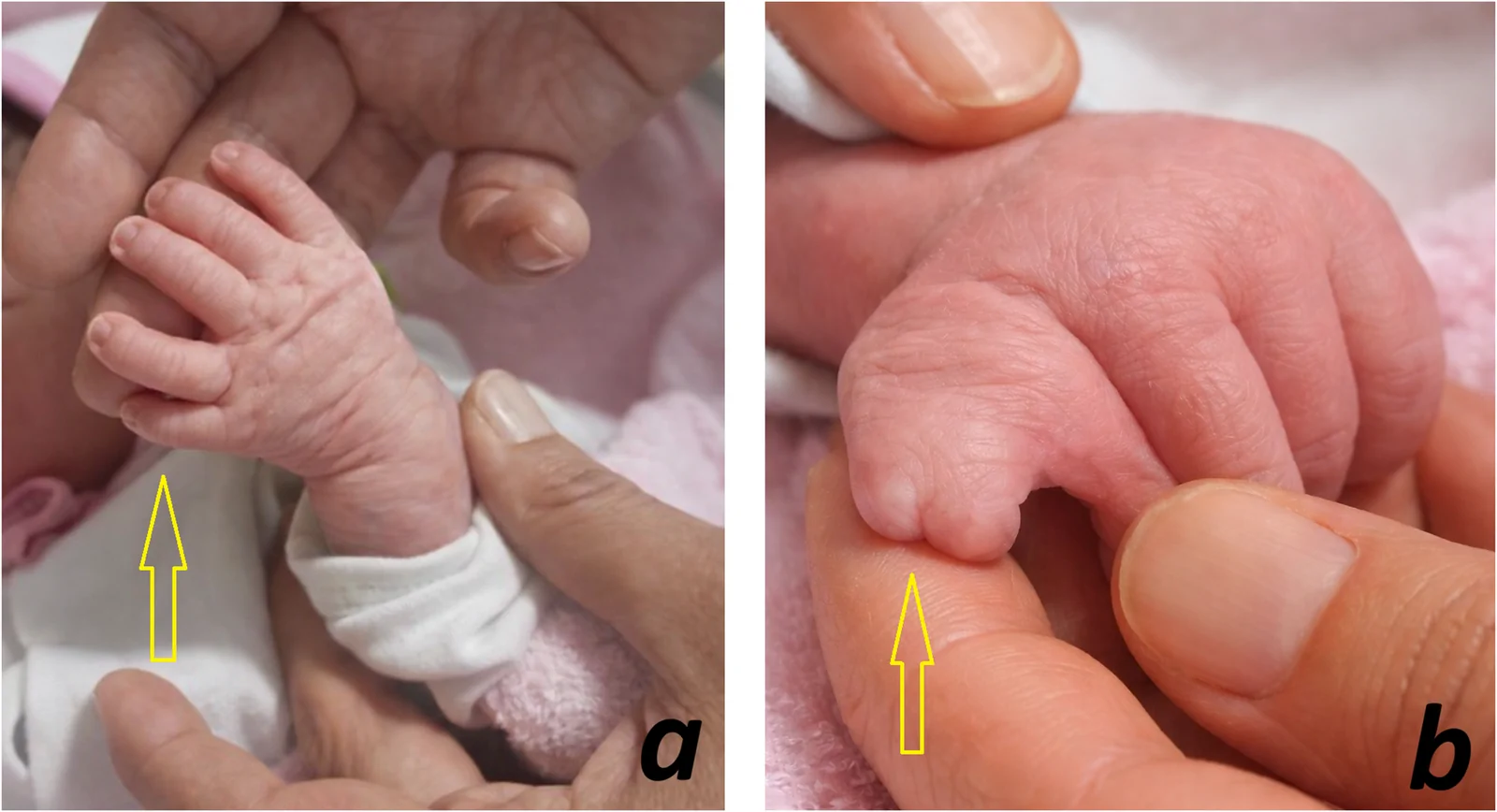
Clinical photograph demonstrating: **(a)** postaxial polydactyly of the left hand in the neonate, showing an additional digit located on the ulnar side. **(b)** Clinical photograph of the right hand showing six digits, with an extra digit fused to an adjacent finger consistent with syndactyly

Craniofacial examination revealed normal ocular and auricular structures with no cleft lip or palate. Limb examination revealed left postaxial polydactyly and right-hand polydactyly with fusion of the supernumerary digit to an adjacent finger (syndactyly) (Fig. 1). Moreover, Perineal examination revealed an anteriorly displaced anus. External genital examination revealed no obvious vaginal bulging or genital mass. A systematic approach based on neonatal emergency principles was undertaken. Initial management focused on stabilization of the airways, breathing, and circulation. Oxygen therapy and intravenous crystalloid fluids were initiated, and her vital signs and urine output were closely monitored until she was stabilized. Given the presence of abdominal distension and feeding intolerance, a nasogastric tube (NGT) was inserted for gastric decompression and subsequent diagnostic evaluations. The initial management was successful, and her general condition improved, yet her abdominal distension and oliguria persisted.

**Fig. 2:**
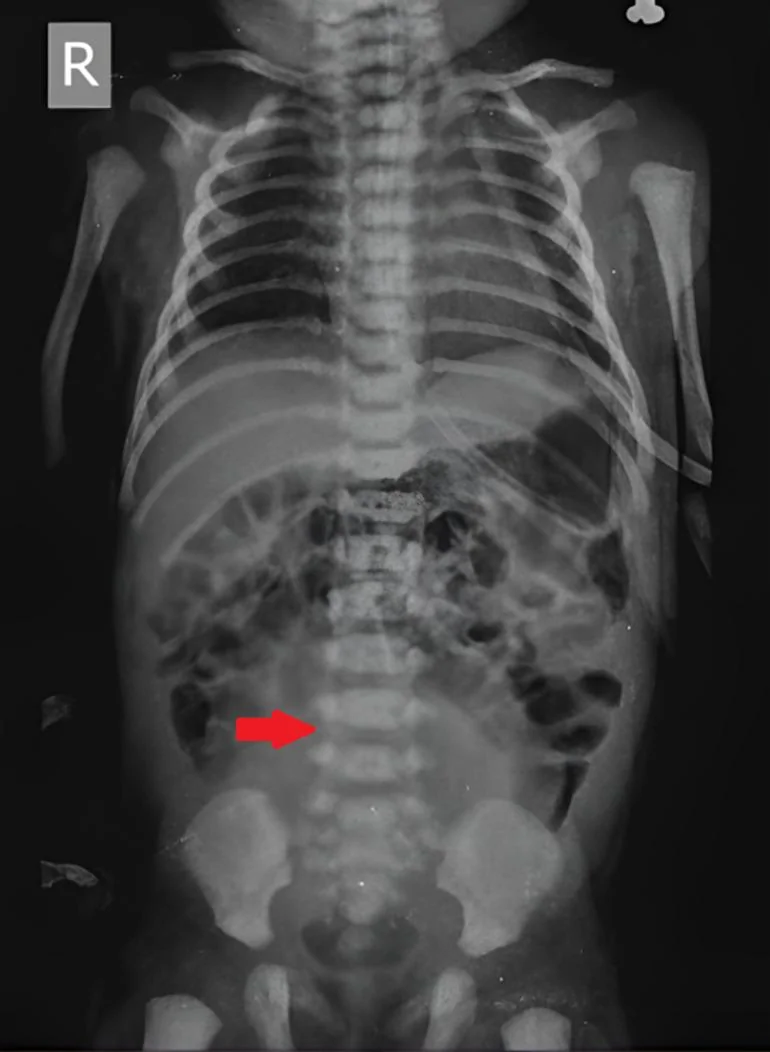
Plain anteroposterior chest X-ray demonstrates a pelvic opacity with mass effect on adjacent intestinal loops

**Fig. 3:**
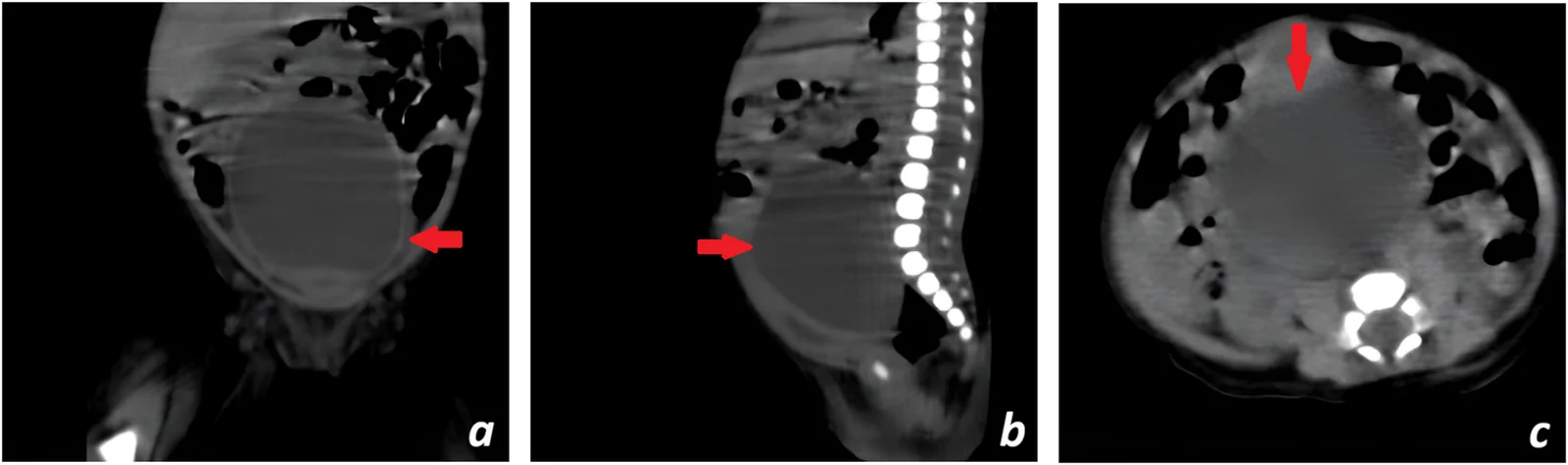
Abdominopelvic computed tomography (CT) showing a cystic lesion measuring 57 × 43 × 64 mm, which is observed superior to the urinary bladder and extends into the pelvis, consistent with hydrometrocolpos (**a**: coronal, **b**: sagittal, **c**: axial views)

Subsequently, the patient was transferred to a tertiary referral hospital for further evaluation. Considering the coexistence of respiratory distress, abdominal distension, and oliguria, differential diagnoses included upper airway obstruction, neonatal intestinal obstruction, sepsis, and renal or metabolic disorders. Laboratory tests were therefore ordered, including complete blood count (CBC), renal function tests, serum electrolytes, blood urea nitrogen, creatinine, blood gas analysis, blood, and urine cultures. Table 1 demonstrates their findings, which were all within normal limits and not suggestive of sepsis or metabolic abnormalities. Following initial stabilization, abdominal radiography was performed, which ruled out the intestinal obstruction (Fig. 2). Subsequent abdominopelvic ultrasonography was conducted to evaluate renal structure, urinary tract patency, and intra-abdominal pathology in the setting of oliguria and abdominal distension. The findings indicated moderate bilateral hydronephrosis with increased renal echogenicity, bilateral compensatory caliectasis suggestive of dysplastic kidneys, and a hydrometrocolpos measuring 32 × 72 mm. An abdominopelvic computed tomography (CT) scan was performed to better define the anatomy for surgical planning. It demonstrated a cystic lesion measuring 57 × 43 × 64 mm located superior to the urinary bladder, extending into the pelvis, consistent with hydrometrocolpos (Fig. 3).

**Table 1:** Laboratory findings on admission

Parameters	Results	Reference Range*
White blood cell count (WBC)	10 × 10³/µL	9–30 × 10³/µL (neonate)
Hemoglobin (Hb)	15.7	14–20 g/dL
Platelet count (PLT)	175 × 10³/µL	150–450 × 10³/µL
Plasma glucose	72 mg/dL	50–120 mg/dL
Creatinine (Cr)	0.3 mg/dL	0.2–0.8 mg/dL
Urea (BUN)	15 mg/dL	5–20 mg/dL
Sodium (Na)	136 mEq/L	135–145 mEq/L
Potassium (K)	4 mEq/L	3.5–5.5 mEq/L
Calcium (Ca)	8.5 mg/dL	8–10.5 mg/dL
Blood gas analysis		
PH	7.38	7.35–7.45
pCO2	40	35–45 mmHg
HCO3	25	18–24 mEq/L
Blood culture	Negative	–
Urine culture	Negative	–

*Reference ranges are typical for term neonates; ranges may vary by laboratory

On the following day, the patient underwent open surgical drainage, during which approximately 50 cc of serous, non-purulent fluid was drained through a cervical incision, and the urachal tract was ligated. Intraoperatively, the rectum was noted to be located anteriorly, adjacent to the vagina, consistent with an anteriorly positioned anus. Rectal patency was confirmed using a Nelaton catheter. Hydrometrocolpos was therefore considered the primary cause of oliguria due to mass effect and compression of the lower urinary tract and bladder outlet, resulting in impaired urinary drainage and secondary bilateral hydronephrosis. The non-purulent nature of the drained fluid and subsequently improved urine output further supported the diagnosis of uterovaginal distension secondary to outflow obstruction.

**Fig. 4:**
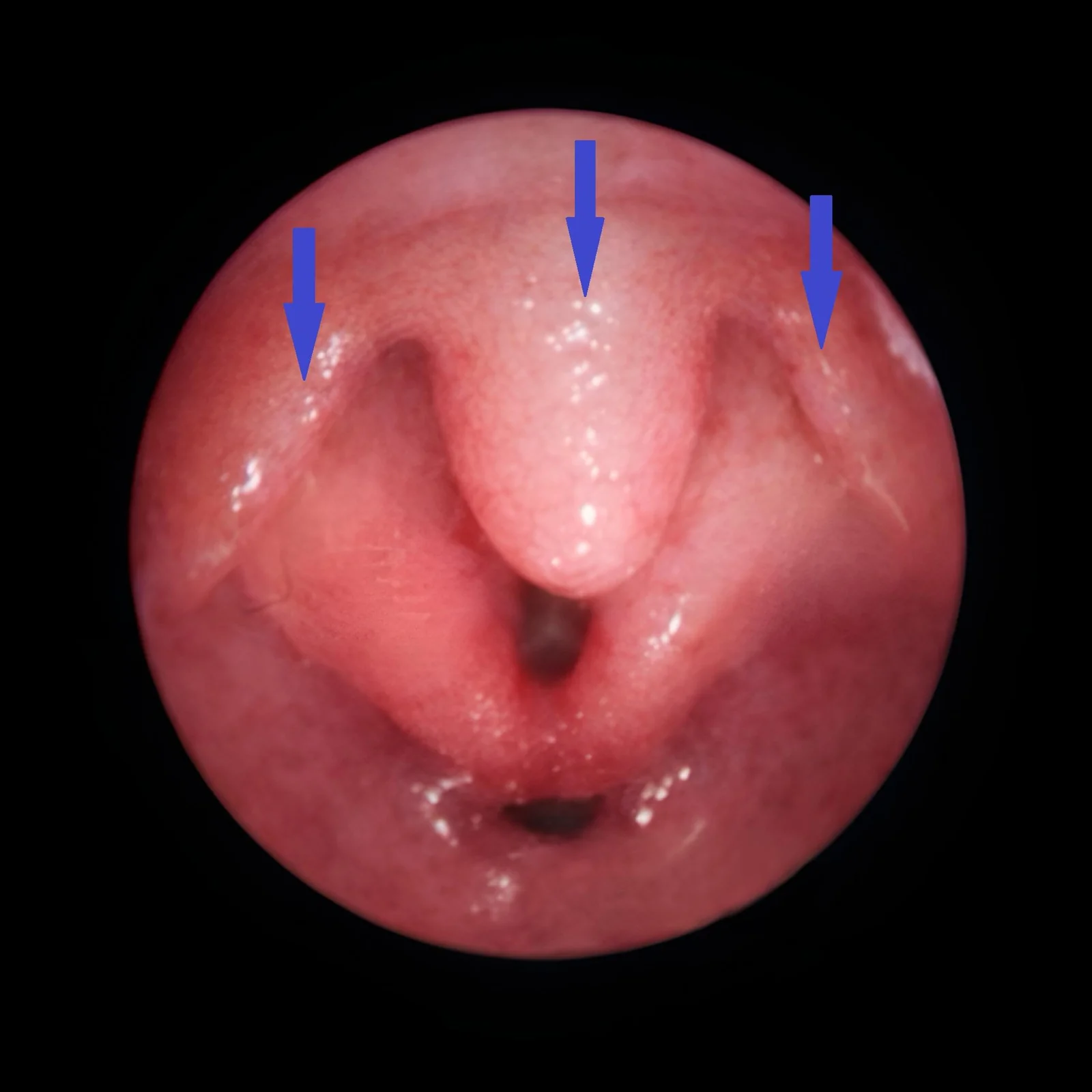
Flexible bronchoscopy image showing a trifid epiglottis (TE) with three distinct epiglottic projections separated by clefts

**Fig. 5:**
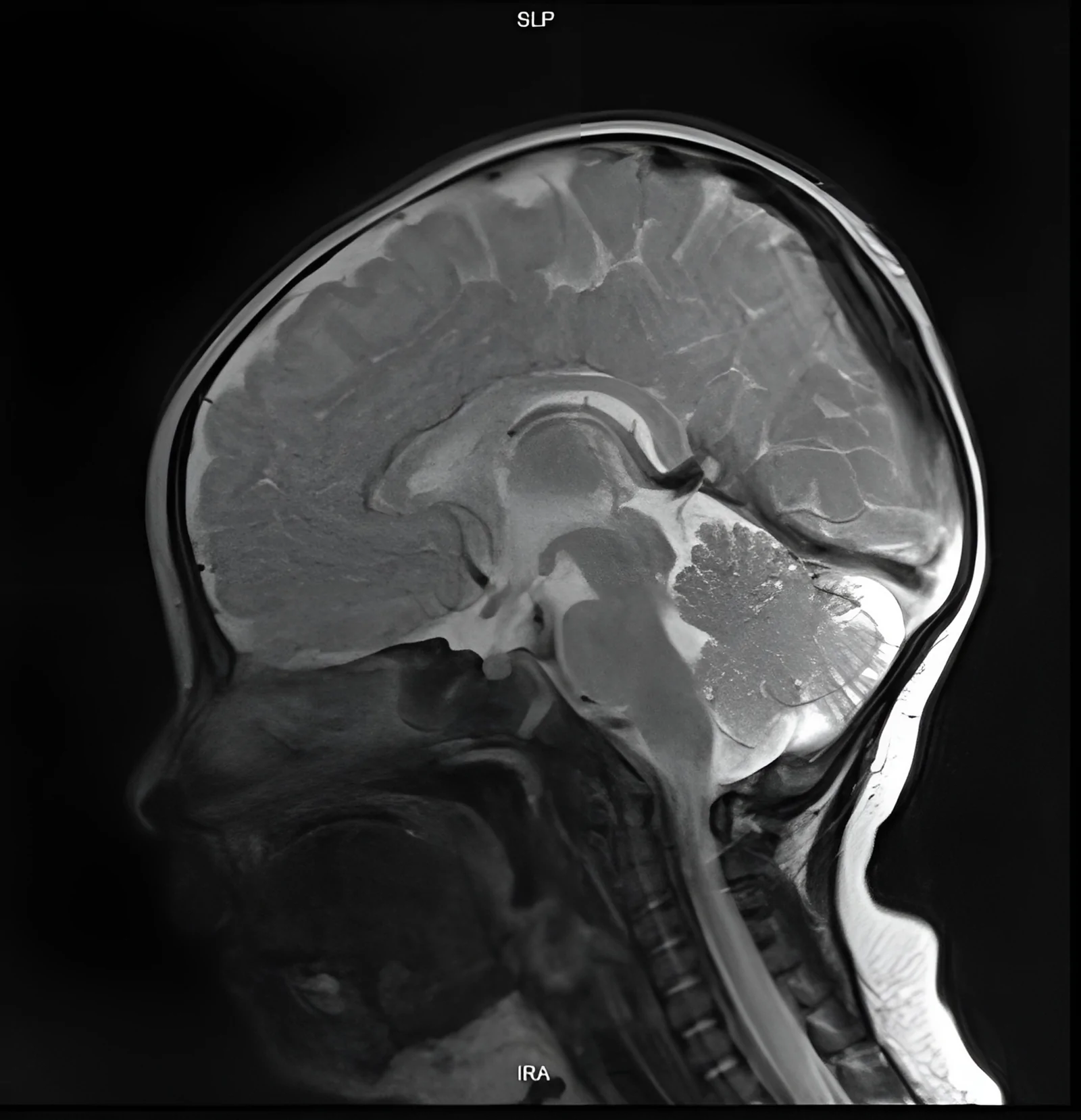
Brain magnetic resonance imaging (MRI) demonstrates normal intracranial anatomy with no evidence of structural abnormalities

Despite improvement in abdominal distension and urine output after surgery, intermittent inspiratory stridor and cyanotic episodes during crying persisted. Therefore, further airway evaluation was performed. Flexible nasal endoscopy confirmed right-sided choanal atresia, which was treated with dilation. Because stridor persisted in spite of choanal dilation, flexible bronchoscopy (FB) was subsequently performed as the definitive airway assessment tool. FB revealed TE along with a grade 1 interarytenoid notch (Fig. 4). Although the vocal cords and trachea appeared normal, both main bronchi were erythematous with thick secretions. Therefore, these secretions were suctioned to relieve the airway obstruction, and these airways were subsequently treated with therapeutic lavage. Additional evaluations were also undertaken to investigate the other co-existing midline anomalies. The echocardiography examination showed no structural heart defects. Spinal ultrasonography revealed a normal spinal canal, spinal cord, and nerve roots, with the conus medullaris in a normal position. No evidence of a tethered cord or space-occupying lesion was noted. Further endocrine evaluation was also performed to assess for pituitary dysfunction. Further endocrine assessments to rule out pituitary dysfunction also exhibited no abnormality (Table 2). A brain magnetic resonance imaging (MRI) also demonstrated no intracranial anomalies (Fig. 5). Because no endocrine or intracranial abnormalities were detected and the patient’s symptoms resolved following surgical management, she was subsequently discharged in stable condition. At one-month follow-up, renal function and respiratory status remained stable, and monthly follow-up up to one year revealed no additional abnormalities.

**Table 2:** Endocrine laboratory results

Parameters	Result	Reference Range (Neonate)*
Random serum cortisol (µg/dL)	14.2	5–25
ACTH (pg/mL) – Adrenocorticotropic Hormone	32	10–60
Free T4 (ng/dL) – Thyroxine	1.3	0.8–2.0
TSH (mIU/L) – Thyroid-Stimulating Hormone	4.1	0.5–6.0
GH (ng/mL) – Growth Hormone	18	5–20
IGF-1 (ng/mL) – Insulin-Like Growth Factor 1	52	30–100
LH (IU/L) – Luteinizing Hormone	1.8	0.4–5.0
FSH (IU/L) – Follicle-Stimulating Hormone	3.2	0.5–5.0

*Reference ranges are typical for term neonates; values may vary by laboratory and age

## Discussion

TE is an extremely rare congenital anomaly of the larynx. To the best of our knowledge, only two cases have been reported in the literature, making the present case the third documented report [4, 5]. Notably, this appears to be the first reported symptomatic neonatal case presenting with clinically significant respiratory manifestations requiring airway evaluation during the neonatal period. Additionally, our patient exhibited several congenital anomalies, including choanal atresia, postaxial polydactyly, an anteriorly displaced anus, and hydrometrocolpos.

The epiglottis develops from the hypobranchial eminence during embryogenesis. Abnormal development or incomplete fusion of these structures may result in epiglottic malformations such as BE, or in extremely rare cases, TE [6]. The coexistence of airway, urogenital, anorectal, and limb anomalies in the present case may suggest disruption of overlapping embryologic developmental pathways involving foregut and mesodermal structures. Developmental abnormalities involving GLI3-mediated signaling pathways have been implicated in syndromic conditions characterized by multisystem malformations affecting the airway, kidneys, genitourinary tract, and limbs, particularly in Pallister–Hall syndrome (PHS) [7, 8]. Notably, the synchronous development of the hypothalamus, epiglottis, and digits might account for the features observed in Bardet-Biedl syndrome (BBS), including polydactyly and other reported midline or developmental anomalies, with rare reports of BE [9, 10]. The characteristic features of these syndromes are summarized in Table 3. These findings suggest that the epiglottic anomalies, whether bifid or trifid, might be a feature of broader syndromic associations. However, due to the extreme rarity of TE, its clinical and genetic associations remain poorly understood. It is worthy to note that one important finding in the present case is that hydrometrocolpos was considered the primary cause of oliguria due to mass effect and compression of the lower urinary tract and bladder outlet, resulting in even secondary bilateral hydronephrosis. Improvement in urine output following drainage further supported this causal relationship, which has been described in previous reports of neonatal hydrometrocolpos [11]. Moreover, hydrometrocolpos has also been described in association with BBS, reflecting its occurrence within broader syndromic multisystem developmental disorders [12].

**Table 3:** Characteristic features of the related syndromes

Syndrome	Key Clinical Presentations
Pallister-Hall Syndrome [13]	Endocrine abnormalities, seizures, polydactyly, bifid epiglottis
Bardet-Biedl Syndrome [10]	Retinal cone-rod dystrophy, facial dysmorphism, polydactyly, obesity, cognitive impairment, hypogenitalism, renal dysplasia, genital anomalies, bifid epiglottis
Joubert Syndrome [14]	Midbrain and hindbrain malformations, developmental delay, hypotonia, breathing abnormalities, syndactyly/polydactyly, atypical eye movements, epiglottic anomalies (bifid epiglottis)

The first case of TE described by Sethi et al. involved a 6.5-year-old child with bilateral polydactyly who developed mild stridor following extubation after surgical removal of extra digits [4]. The second case reported by Sobhan Mishra et al. involved a triad of TE, midline cleft of lip, and polydactyly. In contrast to the first case, the patient was asymptomatic before surgery. Therefore, the anomaly was accidentally detected during intubation for cleft lip repair. The authors highlighted the importance of considering epiglottic anomalies in patients with craniofacial malformations and polydactyly, given the potential challenges that might happen during tracheal intubation, including aspiration and trauma to the anomalous epiglottic structures [5]. In both discussed cases, TE was associated with polydactyly. Similarly, BE is often associated with other anomalies, particularly polydactyly, yet it is more frequently reported compared to TE. Stevens et al. reviewed 22 cases of BE and found that it was never reported as an isolated condition, but almost always part of a syndromic complex [15]. Likewise, Saif et al. reviewed 24 cases of BE reported between 1990 and 2022, identifying nine cases associated with BBS, one with Joubert’s syndrome, six with PHS, and eight cases presenting with various anomalies not related to any known syndrome [10]. In contrast to previously reported cases, our case was symptomatic during the neonatal period and demonstrated a broader spectrum of congenital anomalies, including choanal atresia, an anteriorly displaced anus, and hydrometrocolpos, not just polydactyly. The coexistence of these findings strongly suggests a syndromic condition rather than an isolated anomaly, and the suspicion of midline anomalies was highly increased. Therefore, a brain MRI and endocrine assessments were indicated to fully evaluate the potential intracranial anomalies, which both revealed no abnormalities. The lack of additional systemic findings highlights the heterogeneity of TE presentations and expands its potential clinical spectrum. Moreover, an important clinical finding of this case is the diagnostic role of FB in persistent neonatal stridor. Although choanal atresia may contribute to respiratory symptoms in neonates, persistent stridor should prompt a comprehensive airway evaluation to exclude laryngeal anomalies [16]. In the present case, persistent stridor despite choanal dilation prompted bronchoscopy, which ultimately identified TE and a grade 1 interarytenoid notch. Therefore, this finding highlights the importance of not attributing neonatal stridor solely to more common abnormalities such as unilateral choanal atresia when symptoms persist.

Lastly, from a clinical standpoint, recognition of TE is important to prevent difficulties that may occur in airway management during intubation or anesthesia. Early detection allows clinicians to anticipate potential airway challenges and to implement suitable precautions.

This case had several limitations. One limitation is the lack of genetic testing, which could have provided valuable insights into the potential association of the observed anomalies. Several investigations could have provided further diagnostic clarification, including chromosomal microarray analysis, targeted ciliopathy, and whole exome sequencing [17–20]. Particularly, GLI3 mutations associated with PPS and genes linked to BBS could be considered because both syndromes may present with polydactyly and epiglottic anomalies [21, 22]. Furthermore, the rarity of TE makes it difficult to draw a definitive conclusion about its clinical spectrum. Despite these limitations, this case provides valuable clinical insight because, based on our knowledge, it is the first reported neonatal presentation of symptomatic TE and broadens the range of associated congenital anomalies. Further case reports and genetic investigations are needed to better understand the embryological mechanisms and possible syndromic associations of TE.

## Conclusion

To the best of our knowledge, this report describes the first reported neonatal presentation of symptomatic TE associated with polydactyly, a combination that is only documented twice. Additionally, this patient presented with other rare associations, such as choanal atresia, hydrometrocolpos, and an anteriorly displaced anus. The case highlights the importance of comprehensive airway evaluation and a systemic approach in neonates presenting with multiple congenital anomalies. Early detection of such anomalies may help anticipate airway challenges and guide multidisciplinary management for long-term follow-up.

## Data Availability

All data generated or analyzed during this case report are included in this published article.

## Abbreviations

TE: Trifid Epiglottis
BE: Bifid Epiglottis
BBS: Bardet–Biedl Syndrome
PHS: Pallister–Hall Syndrome
MRI: Magnetic Resonance Imaging
CT: Computed Tomography
NGT: Nasogastric Tube
CBC: Complete Blood Count
SpO₂: Peripheral Oxygen Saturation
G3P3: Gravida 3, Para 3
Flexible bronchoscopy: FB
